# 

**Published:** 1923-01

**Authors:** 


CONTENTS.

*Page*

The Debt of Medicine to the Fine Arts. By J. A. NIXON, C.M.G., M.D. Cantab., F.R.C.P. ... 1

The Principles of Surgical Treatment of Infections of the Peritoneum. By FORBES FRASER, C.B.E., F.R.C.S. ... 29

Acquired Resistance to Tuberculosis: a Factor in Clinical Type and Prognosis. By S. LYLE CUMMINS, C.B., C.M.G., M.D. ... 41

Critical Review ... 52

Reviews of Books ... 58

Editorial Notes ... 66

Meetings of Societies ... 67

Obituary: J. M. RATTRAY, M.A., M.D. ... 69

Local Medical Notes ... 70

